# Clinical Features and Management of Suboptimal Ovarian Response During *in vitro* Fertilization and Embryo Transfer: Analysis Based on a Retrospective Cohort Study

**DOI:** 10.3389/fendo.2022.938926

**Published:** 2022-07-22

**Authors:** Yizhi Yan, Ruomu Qu, Xiaodong Ma, Siyuan Qin, Lixue Chen, Xiaoxiao Ni, Rui Yang, Ying Wang, Rong Li, Jie Qiao

**Affiliations:** ^1^ Department of Obstetrics and Gynecology, Reproductive Medical Center, Peking University Third Hospital, Beijing, China; ^2^ Peking University Health Science Center, Beijing, China

**Keywords:** controlled ovarian hyperstimulation, *in vitro* fertilization & embryo transfer, suboptimal ovarian response, super-long protocol, long protocol, antagonist protocol, retrospective cohort study

## Abstract

**Background:**

Based on dynamic changes of indicators during controlled ovarian hyperstimulation and of clinical outcomes of suboptimal ovarian response with different protocols, this study aimed to summarize the clinical characteristics of SOR and provide clinical recommendations.

**Methods:**

Data of 125 patients with SOR and 125 controls who had undergone appropriate protocols for *in vitro* fertilization-embryo transfer were collected from a single medical center from January 2017 to January 2019. Basic clinical indexes, including age, BMI, antral-follicle count, infertility time, basic follicle-stimulating hormone, luteinizing hormone, LH/FSH ratio, estradiol, progesterone, testosterone, androstenedione, prolactin, anti-Mullerian hormone, and thyroid stimulating hormone levels, were analyzed using T-test. Dynamic indexes during COH, including amount and days of gonadotropin, sex hormone levels, and number of large/medium/small follicles at specified time periods, were analyzed using T-test and joint diagnosis analysis with ROC curves. Indexes of laboratory and clinical indicators were analyzed using the chi-square test.

**Results:**

For the SOR group, BMI, duration time, and dosage of gonadotropin used for SOR were significantly higher. In the ultra-long/long group, ROC curve analysis showed that the LH/FSH ratio and BMI yielded cutoff values of 0.61 and 21.35 kg/m^2^, respectively. A combined diagnosis of the two indexes showed higher sensitivity (90%) and specificity (59%). In the GnRH-ant group, ROC curve analysis showed an LH level, an LH/FSH ratio on COH day 2, and BMI yielded cutoff values of 2.47 IU/L, 0.57, and 23.95 kg/m^2^, respectively. Combining the two indexes with BMI, both showed increased sensitivity (77%) and specificity (72% and 74%). The estradiol level and progesterone level during the late follicular stage in SOR patients were significantly lower than those in control patients for both protocol groups. At each monitoring time, delayed follicular development was observed. The live-birth rate in fresh cycles of the ultra-long/long group and the live-birth rate in cumulative cycles of the antagonist group in the SOR group were lower than those in the control group.

**Conclusion:**

SOR had adverse effects on clinical outcome. We provide some threshold values of basic LH/FSH ratio, BMI, COH day 2 LH, counts of follicles, and levels of estradiol/progesterone to be taken as reference to assist the early recognition of SOR.

## 1 Introduction

In controlled ovulation hyperstimulation (COH) during *in vitro* fertilization-embryo transfer (IVF-ET), the optimal selection of an ovulation hyperstimulation protocol is one of the key factors affecting the success rate of IVF. During the IVF process, most patients undergo normal reactions, which have not been clearly defined. According to experts, indicators, including age, ovarian reserve function, and past history of COH (low or high reaction), could be used to comprehensively evaluate ovarian reactions. Women with pure oviduct factors and/or male infertility belong to the population with normal ovarian reactions. Specific indicators predicting ovarian normal reaction (1) include age <35 years, normal ovarian reserve function, 1.0–1.4 g/l < anti-Mullerian hormone (AMH) <3.5–4.0 g/l, 7 < antral follicle counts (AFC) <14, follicle-stimulating hormone (FSH) level <10 IU/l, and no previous cancelled cycles due to low or high ovarian response. The most suitable ovum number after IVF is 5–15, with a high ovum maturity rate and high quality, which can achieve better clinical outcomes after IVF.

**Figure 1:**
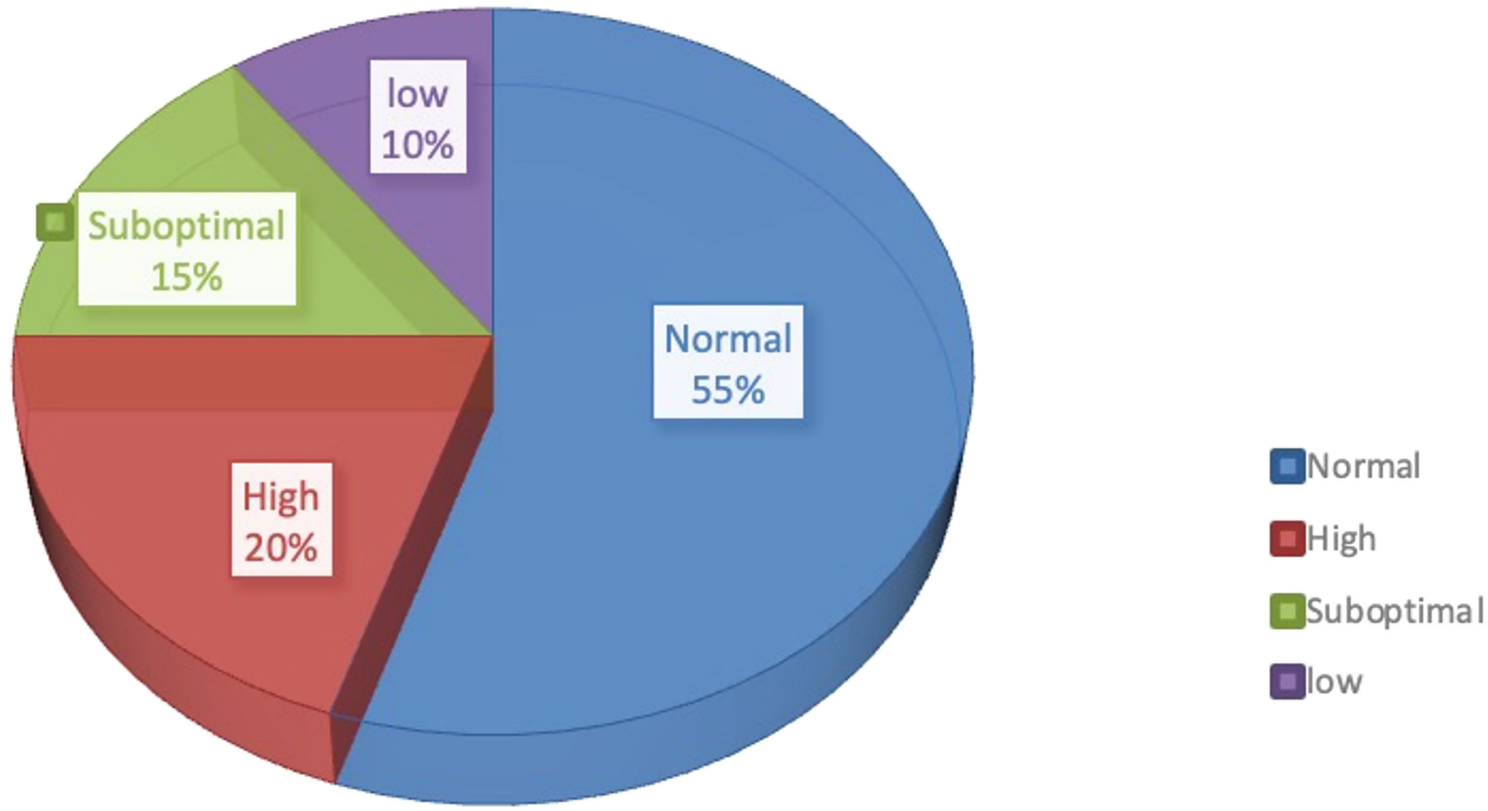
Proportion of different ovarian responses during controlled ovarian responses types in IVF.

In addition to the normal response, a considerable proportion of patients showed high and low ovarian responses, accounting for approximately 20% and 10%, respectively (**Figure 1**) (2). High ovarian response refers to an abnormal sensitivity of the ovary to gonadotrophin (Gn) stimulation during COH. Currently, no unified diagnostic criteria toward high ovarian response have been established. Previous reports mostly define high ovarian response as acquired ovum number >15 or estradiol (E2) peak >3,000 pg/ml (3), which mainly occurs in patients with PCOS, patients with low body weight or low BMI, or patients with a previous history of high ovarian response. High estrogen levels lead to increased vascular permeability and eventual extravasation of blood into the third body cavity (4). As a result, varying degrees of hydrothorax, ascites, and cerebral edema, as well as varying degrees of blood concentration, hypovolemic shock, and/or venous thrombosis, may occur with severe consequences. Low ovarian response refers to the decreased ability of cortical follicles to grow, develop, or be fertilized to form embryos. The characteristics of poor ovarian response to Gn stimulation during the COH process include decreased developing follicle number, decreased peak of serum E2 level on the day of human chorionic gonadotropin (HCG) administration, increased use of Gn, decreased acquired ovum number, and poor clinical pregnancy rate (5). Patients with a low ovarian response usually have advanced age or normal age (less than 35-year old) with poor ovarian reserve function. Additionally, another ovarian response type, suboptimal ovarian response (SOR), has similar clinical manifestations but different mechanisms from low ovarian response. About 10%–15% of patients using gonadotropin-releasing hormone agonist (GnRH-a) exhibit SOR after downregulation of the pituitary gland (6). Different from low ovarian response, SOR is not induced by a decline in ovarian reserve function. Patients with SOR have normal age (less than 35 years old) with normal sex hormone levels, AMH level, and AFC. However, they exhibit abnormally slow follicular growth in the IVF process. If SOR is recognized in time and managed with appropriate remedial measures, it could still be converted into normal ovarian response, with optimal number of acquired ova (7). The diagnostic criteria of SOR are as follows: 1) no presence of follicles with a diameter >10 mm on the 6th–8th days of ovarian stimulation; 2) serum E2 level <655.1–728.3 pmol/l on the 6th day of ovarian stimulation; 3) slow follicular development and increased follicular diameter <3 mm within 3 days (1).

Currently, several studies have been conducted on SOR, but its mechanism of occurrence and related factors remain elusive. Additionally, its effect on COH and pregnancy outcomes is inconclusive. SOR may have a negative effect on the outcome of ovulation induction and pregnancy (8). Since SOR can be easily ignored, inappropriate handling of SOR cases may lead to a transition to low ovarian response with higher risk of cycle cancellation and increase in patients’ economic and psychological burdens. In this study, a retrospective cohort analysis was performed on the clinical data of patients with SOR and normal ovarian response from January 2017 to January 2019. These patients must have undergone procedures following the ultra-long protocol, long protocol, and antagonist protocol for IVF-ET at the Reproductive Center of the Peking University Third Hospital. Additionally, the study explored the influencing factors on SOR, predictive value of indicators, and effect of SOR on the outcome of ovulation induction and clinical pregnancy outcome. Findings from this study may establish a theoretical basis and data summary for improving the poor outcomes of SOR cases.

## 2 Materials and methods

### 2.1 Patients

**Figure 2:**
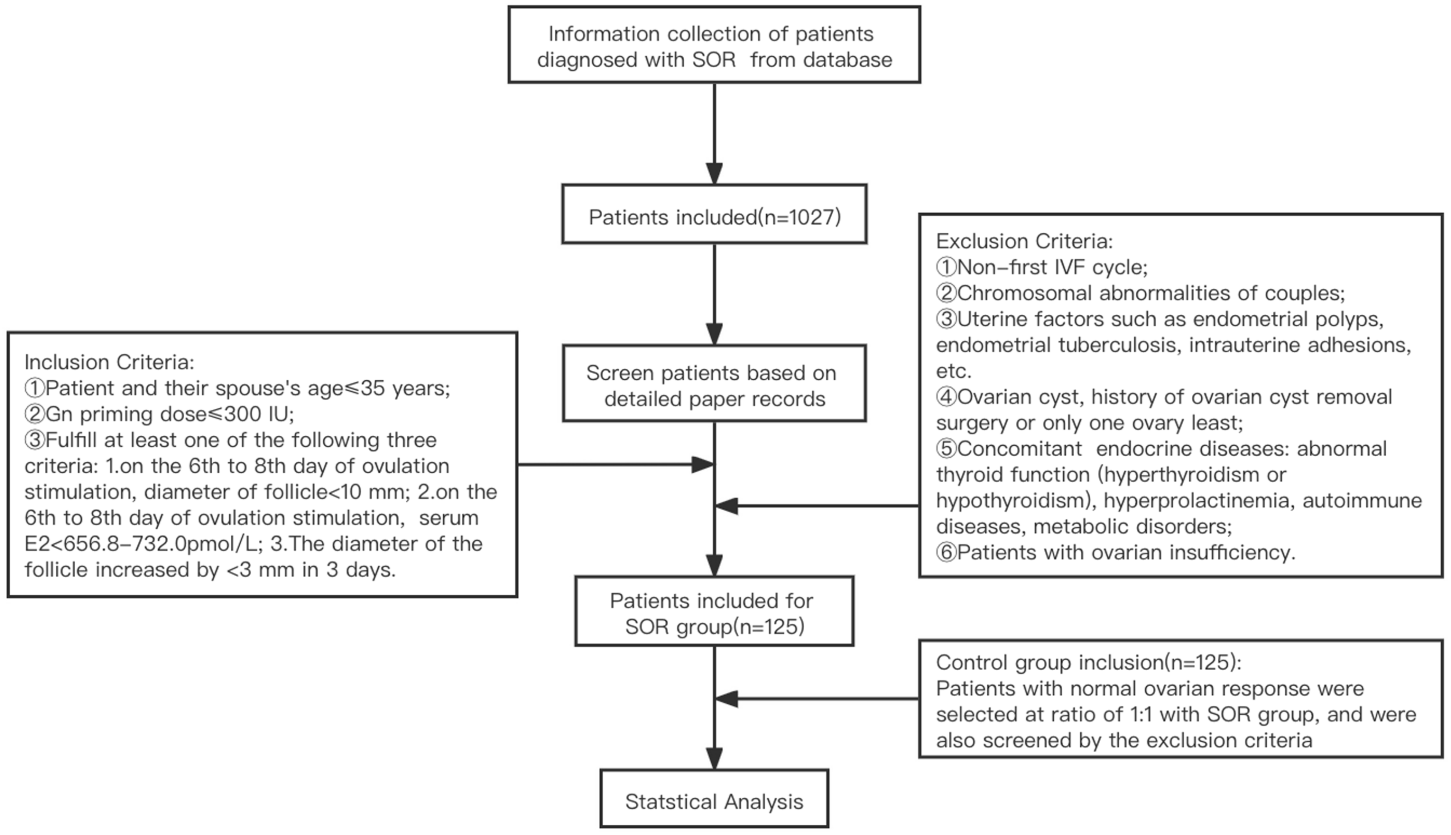
Process of the Patients Recruitment.

A retrospective analysis was performed on clinical data from 125 patients with SOR (slow growth of follicles during COH and low serum E2 level, based on diagnostic criteria of SOR talked above) who underwent IVF-ET at Peking University Third Hospital from January 2017 to January 2019. According to the COH ovarian stimulation protocol, they were divided into ultra-long and long protocol group (71 cases) and antagonist protocol group (54 cases). Due to the limited sample size and similarity between the mechanisms of the ultra-long protocol and long protocol, both groups were combined into a single group for the analysis. The control group consisted of 125 patients with normal ovarian response who had undergone IVF-ET during the same period as the SOR group, with a similar number of patients in the corresponding protocol group in the SOR group. Due to attrition resulting from patients who canceled the cycle, some medical records were incomplete. Therefore, canceled cycles were not analyzed in this study. An overview of the patient recruitment process is shown in **Figure 2**.

### 2.2 IVF-ET Treatment Protocol

Generally, evaluation of ovarian function is based on age, weight, AFC, serum level of AMH, and basal serum level of hormones, followed by the development of COH protocols. Commonly used drugs during standardized long or ultra-long protocols include GnRH-agonist (GnRH-a) and Gn, while those during antagonist protocol are Gn and GnRH-antagonist (GnRH-ant). GnRH-a mainly used in our center include Diphereline (Ipsen Pharma Biotech, Signes, France) or Enantone (Leuprorelin, Takeda Pharma Company, Tokyo, Japan), and Gn used in our center include 1) recombinant FSH: Gonal-F (Merck Serono, Geneva, Switzerland), Puregon (Organon, P.O. Box 20 OssNL5340BH, Netherlands), and Urofollitropin (uFSH, Livzon Pharmaceutical Group Inc., Zhuhai, China); 2) menotropins for injection (urine-derived HMG, Livzon Pharmaceutical Group Inc., Zhuhai, China) and Menopur (high-purity urine-derived HMG, Ferring GmbH, Kiel, Germany); and 3) recombinant LH: Luveris (Merck Serono S.A). Antagonists used in our center include cetrorelix acetate (Pierre Fabre Pharmaceuticals, Castres, France) and Ganirek (N.V. Organon, Nijmegen, Netherlands).

Recombinant HCG (Merck Serono SA Aubonne Branch) was injected to retrieve oocytes. COH protocols refer to expert consensus on assisted reproduction ovulation induction drug treatment (9). The treatment process of each protocol, the exact drugs used during process, and the standards for pituitary regulation are described in Appendix E1 and Appendix E2.

### 2.3 Oocyte Retrieval and Transplantation

#### 2.3.1 The Timing of Oocyte Retrieval

The dose and type of Gn were adjusted based on the patient’s age and follicular development. The patient’s level of serum LH, E2, and P and diameter of the follicles were monitored. When the patient’s dominant follicles’ diameter reached ≥18 mm, 250 μg of HCG (Shanghai Livzon Pharmaceutical) or Ovidrel (recombinant HCG, Merck Serono SPA) was injected intramuscularly. Thirty-six hours later, the oocytes were retrieved under vaginal ultrasound guidance. IVF or intracytoplasmic sperm injection (ICSI) fertilization was chosen based on the condition of the semen.

#### 2.3.2 Embryo Transfer

The embryos transferred were clinically transferable embryos, and the embryo transfer program included the cleavage embryo transfer and blastocyst transfer. The Reproductive Medical Center of Peking University Third Hospital assessed the morphology of cleavage embryos based on the number of blastomeres for uniformity in size and fragmentation of the blastomeres. The embryos could be used with at least four cells and cell fragments below 30%. The blastocyst-stage embryo scoring used the blastocyst grading system proposed by Gardner et al. (10). According to the size of the blastocyst cavity and whether it was hatched, the development of the blastocyst was divided into six stages: stage I (early stage, blastocyst with cavity, the volume of the embryo cavity was less than one-half of the total volume); stage II (the volume of the blastocyst cavity was ≥1/2 of the embryo volume); stage III (the blastocyst cavity completely occupied the total volume of the embryo); stage IV (the blastocyst expanded, the blastocyst cavity when the embryo was completely occupied, the total volume of the embryo became larger, and the zona pellucida became thin); stage V (the blastocyst was hatching, part of the blastocyst escaped from the zona pellucida [ZP]); and stage VI (the blastocyst was hatched, completely escaped from the ZP). Blastocysts in stages IV and V and some embryos in stage VI (including VIBC, VICB) could be used for embryo transfer.

Our center routinely performed fresh embryo transfer on day 3 post-fertilization. In the presence of factors that were not suitable for fresh embryo transfer, such as high risk of ovarian hyperstimulation syndrome (OHSS), endometrial factors, uterine effusion, progesterone elevation, and personal factors, the transfer would be cancelled, and remaining embryos or blastocysts would be vitrified and frozen, and thawed embryo transfer (FET) would be performed at an optimal time.

#### 2.3.3 Follow-Up After Transplantation

Luteal phase support would be provided routinely after embryo transfer, involving oral, vaginal, or intramuscular progesterone. A blood test was performed after 14–16 days of transplantation. Serum HCG >5 mIU/ml was defined as a positive result (the detection of blood HCG in the Endocrinology Laboratory of Peking University Third Hospital Reproductive Medical Center adopts double-site enzyme immunoassay (double-antibody sandwich) kit from Beckman Coulter, Brea, CA, USA). Biochemical pregnancy referred to the pregnancy in which the blood HCG was positive, but the gestational sac echo was not detected by ultrasound. When serum HCG levels were elevated, transvaginal ultrasound was performed about 4–5 weeks after embryo transfer, and the pregnancy sac was found in or outside the uterine cavity, clinical pregnancy was diagnosed. In the absence of pregnancy, corpus luteum support was stopped. If intrauterine pregnancy was confirmed, luteal support was continued until 9–10 weeks of pregnancy.

### 2.4 Indicators for Further Observation

#### 2.4.1 General Information of Patients

Age, age of spouse, body mass index (BMI), AFC, years of infertility, basic FSH, basic LH, basic E2, basic pituitary prolactin (prolactin, PRL), basic testosterone (T), basic androstenedione (AND), basic thyroid stimulating hormone (TSH) levels, levels of AMH, LH, FSH, E2, and P on 2nd day of COH, and vaginal ultrasound were used to detect the number of ovarian antral follicles. Follicle sizes were defined as follows: minimum follicle, diameter ≤10 mm; small follicle, 10 mm < diameter ≤15 mm; medium follicle, 15 mm < diameter of follicle ≤18 mm; large follicle, diameter >18 mm. Hormone levels were measured by electrochemiluminescence (Roche Diagnostics, Mannheim, Germany), and the operation was performed according to the manufacturer’s instructions.

#### 2.4.2 Indicators of Ovulation Induction

Gn using days; Gn using dose; COH 2nd-day FSH, LH, P, and E2 levels; changes in follicle number and diameter; LH, P, and E2 levels on COH 6th–8th, 9th–11th, 12th–14th, and 15th–17th days, and others; P/number of large follicles (progesterone to follicle index, PFI); and P/E2 ratio on HCG day were the indicators of ovulation induction.

#### 2.4.3 Laboratory Indicators of Ovulation Outcome

These indicators included number of oocytes obtained, 2PN oocytes, fertilization number, cleavage number, high-quality embryo number, and corresponding 2PN rate, fertilization rate, cleavage rate, and high-quality embryo rate.

#### 2.4.4 Indicators of Pregnancy Outcome

They included biochemical pregnancy rate, clinical pregnancy rate, live birth rate, abortion rate, and term birth rate. As the data of patients whose cycles were cancelled due to SOR in our center were lost to follow-up due to the patients’ personal reasons, the statistics of SOR incidence and cycle cancellation rates were not accurate. Therefore, these two indicators were not calculated.

### 2.5 Statistical Analysis

All statistical analyses were performed using SPSS 24.0 (IBM SPSS Statistics CRZ1AML for Windows). Measurement data were expressed as mean ± standard deviation, and enumeration data were expressed as rates (%). Measurement data were tested for normality (P > 0.05). The data were normally distributed; therefore, t-test was used. The enumeration data were assessed using the chi-square test. The ROC survival curve was used to calculate the cutoff value, and the unconditional binary regression model was used for multivariate analysis. P < 0.05 indicated that the difference was statistically significant.

## 3 Results

### 3.1 Baseline Information of All Patients

Among patients enrolled, the baseline data of patients in the ultra-long and long protocol group were compared separately, and no statistical difference was observed (P > 0.05) (**Supplementary Table 1**). Therefore, the two groups were combined into the ultra-long/long group for subsequent analyses. In the ultra-long/long group, compared with the control group, the SOR group had a higher BMI (P < 0.05) and higher AMH (P < 0.05); in the antagonist group, the SOR group was observed to have a higher BMI (P < 0.05) and lower TSH. No difference was observed between SOR patients and control patients in both protocol groups in age, spouse age, years of infertility, types of infertility, basal sex hormone levels, and basal follicle number (P > 0.05) (**Table 1**).

**Table 1:** Baseline information of all patients in IVF.

Index	Ultra-long/long protocol			Antagonist protocol		
	SOR group(n = 71)	Control group (n = 71)	*P value*	SOR group (n = 54)	Control group (n = 54)	*P value*
Age	29.68 ± 2.94	30.41 ± 3.18	0.156	28.87 ± 3.42	29.04 ± 3.08	0.791
Spouse age'	30.65 ± 2.88	31.08 ± 2.99	0.377	29.46 ± 2.85	29.67 ± 3.09	0.722
Infertility year	2.90 ± 2.22	3.04 ± 1.93	0.688	3.57 ± 2.55	3.41 ± 2.81	0.748
Primary infertility	41	47	0.300	34	33	0.843
Secondary infertility	30	24		20	21	
BMI(kg/m^2^)	24.75 ± 3.47	21.64 ± 3.11	0.000	26.31 ± 3.47	23.52 ± 3.94	0.001
Basal FSH(IU/l)	6.56 ± 2.50	6.08 ± 2.01	0.240	6.19 ± 1.78	5.88 ± 1.68	0.406
Basal LH(IU/l)	3.93 ± 2.34	4.65 ± 2.73	0.116	5.59 ± 5.19	5.67 ± 3.35	0.073
Basal E2(pmol/l)	170.11 ± 110.02	172.24 ± 65.49	0.896	171.55 ± 82.43	202.32 ± 267.25	0.468
Basal PRL(ng/ml)	24.17 ± 45.30	17.95 ± 42.04	0.438	11.15 ±4.18	26.09 ± 61.12	0.113
Basal T(nmol/l)	2.85 ± 9.22	1.22 ± 3.55	0.220	2.89 ± 9.50	4.67 ± 11.42	0.431
Basal AND(nmol/l)	7.16 ± 3.51	5.98 ± 2.77	0.060	7.18 ± 3.63	8.31 ± 4.92	0.249
AMH(ng/ml)	4.76 ± 3.19	3.41 ± 2.32	0.013	5.29 ± 3.48	5.03 ± 4.06	0.769
TSH(µIU/ml)	2.54 ± 1.74	2.34 ± 1.33	0.465	2.12 ± 0.77	3.21 ± 2.18	0.001
AFC(bilateral)	13.39 ± 4.87	11.98 ± 5.45	0.117	16.13 ± 6.71	15.58 ± 5.74	0.653

### 3.2.1 Comparison of Indexes in the COH Process

#### 3.2.1 Ultra-Long/Long Group Population

##### 3.2.1.1 Univariate Analysis

**Figure 3:**
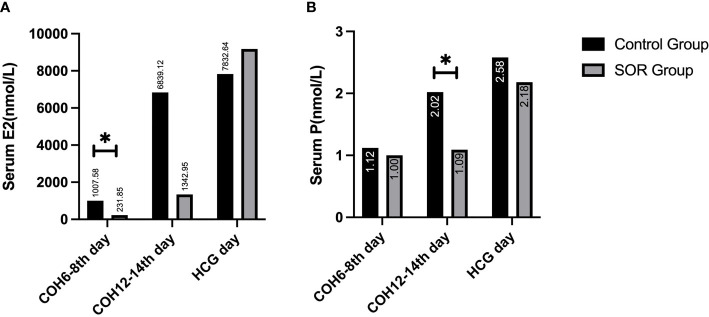
**(A)** Comparison of serum E2 levels during the COH process in the ultra-long/long group; **(B)** Comparison of serum P levels during the COH process in the ultra-long/long group (note: (1).*Indicates statistically significant difference (P < 0.05); (2) n (SOR group) = 71, n (control group) = 71).

In the ultra-long and long protocol group, the SOR group had a longer response time to downregulation and longer Gn application time than the control group, and their total dose of Gn using was more than that of the control group. The basic LH/FSH ratio of the SOR group was 0.63 ± 0.37, significantly lower than the value of 0.84 ± 0.65 in the control group (P < 0.05) (**Supplementary Table 2**). The serum E2 level of COH on days 6–8 was 231.85 ± 108.58 pmol/l, significantly lower than the value of 1007.58 ± 1002.80 pmol/l in the control group (**Figure 3A**), and the serum P level of COH on days 12–14 was 1.09 ± 0.55 nmol/l, significantly lower than the value of 2.02 ± 1.11 nmol/l in the control group (P < 0.05) (**Figure 3B**) (**Supplementary Table 2**). On HCG day, the PFI and P/E2 ratios in the SOR group were 0.59 ± 0.25 and 0.33 ± 0.24 respectively, higher than those in the control group (0.56 ± 0.41 and 0.12 ± 0.68, respectively), but without statistical significance (**Supplementary Table 2**).

**Figure 4:**
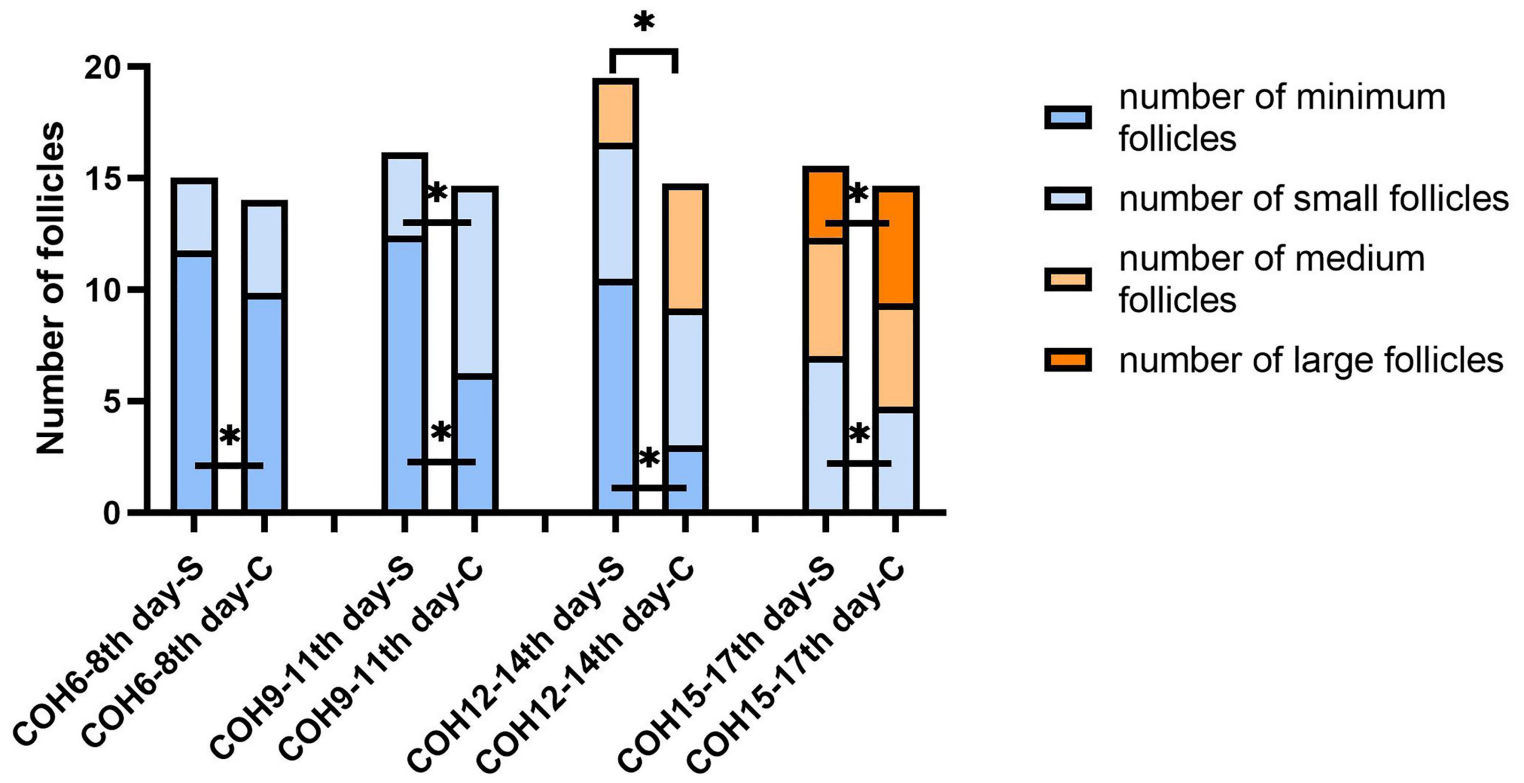
Changes in the number of follicles of different sizes during the COH process in the ultra-long/long group patient (note (1): *Indicates statistically significant difference (P < 0.05); (2) S:SOR group; C: control group; (3) n (SOR group) = 71, n (control group) = 71).

The minimum follicle number in the SOR group on days 6–8, 9–11, and 12–14 of COH were 11.78 ± 4.29, 12.42 ± 5.15, and 10.49 ± 4.91 respectively, all higher than the values in the control group (9.86 ± 4.63, 6.24 ± 3.02, 3.00 ± 1.41, respectively) with significance (P < 0.05). The number of small follicles in the SOR group on days 15–17 of COH was 7.03 ± 4.21, significantly higher than that of the control group, which was 4.75 ± 1.73 (P < 0.05), whereas the number of small follicles on days 9–11 of COH in the SOR group, the number of medium follicles on days 12–14 of COH in the SOR group, and the number of large follicles on days 15–17 of COH in the SOR group were lower than those in the control group (P < 0.05)(3.75 ± 3.26 vs. 8.42 ± 3.85, 2.89 ± 1.90 vs. 5.60 ± 3.53, and 3.24 ± 2.09 vs. 5.27 ± 1.49, respectively. Shown in **Figure 4**). Other details are included in **Supplementary Table 2**. Some of the indicators on certain measurement dates were not included in the statistical comparison due to the limited number of recorded medical records.

##### 3.2.1.2 ROC Curve Analysis and Joint Diagnosis

Since the early identification and remediation of SOR is important, this study used the ROC curve to analyze the relatively early (COH ≦14 days) indicators with statistical differences between the SOR group and control group in each protocol group.

In the univariate analysis, the data with statistical differences in the early follicular phase and the median follicular phase were analyzed using the ROC curve according to the COH time sequence. Additionally, the threshold of SOR occurrence and the corresponding sensitivity and specificity of each index were calculated. The results are shown in **Table 2**. The combined diagnostic analysis of BMI and basal LH/FSH ratio showed that sensitivity increased to 90% and specificity was 59% (AUC, 0.814; 95% Cl, 0.738–0.889).

**Table 2:** Results of single-factor indexes during the COH process in the ultra-long/long group patients.

Time	Index	AUC	Threshold	Sensitivity	Specificity	95% confidence interval
BMI (kg/m^2^)		0.722	21.35	87%	35%	0.695-0.850
Basal LH/FSH ratio		0.631	<0.61	68%	63%	0.532-0.731
COH days 9–11	Number of minimum follicles	0.847	>10.5	72%	93%	0.763–0.931
	Number of small follicles	0.822	<3.5	63%	95%	0.693–0.951
COH days 12–14	Level of serum P	0.846	<1.52 nmol/L	95%	65%	0.734–0.959
	Number of minimum follicles	0.969	>5.5	93%	100%	0.923–1.000
	Number of medium follicles	0.760	<2.5	56%	82%	0.605–0.914

#### 3.2.2 Antagonist Group Population

##### 3.2.2.1 Univariate Analysis

**Figure 5:**
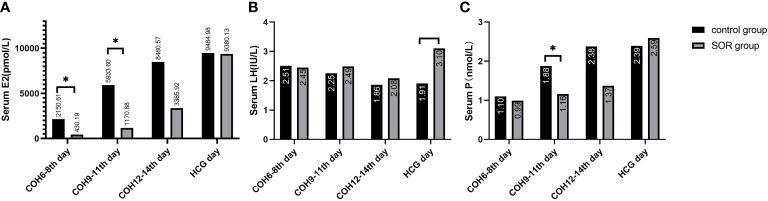
**(A)** Level of serum E2 during the COH process in the antagonist group; **(B)** Level of serum LH during the COH process in the antagonist group patient; **(C)** Level of serum P during the COH process in the antagonist group patient (note: (1) *Indicates statistically significant difference (P < 0.05); (2) n (SOR group) = 54, n (control group) = 54).

**Figure 6:**
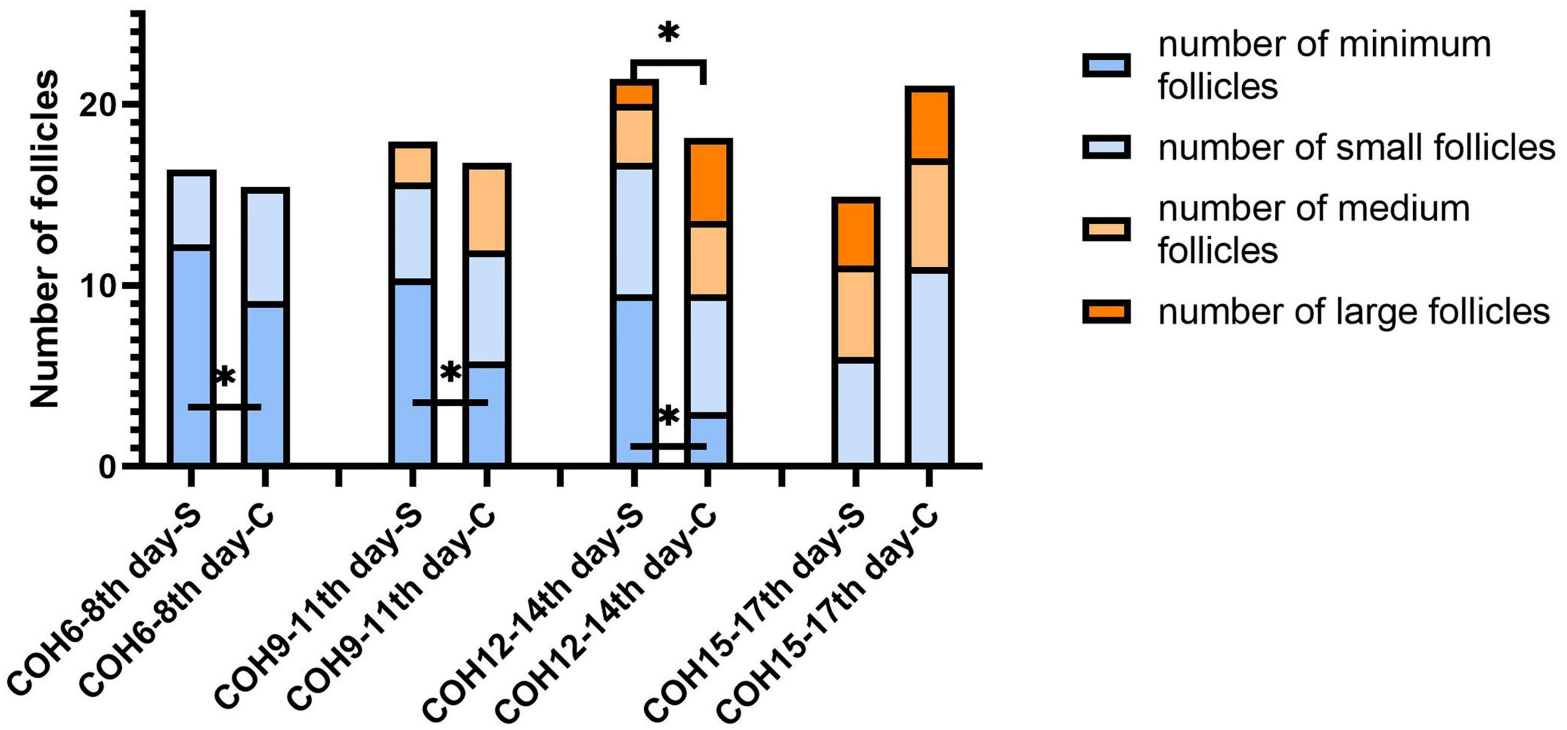
Changes in the number of follicles of different sizes during the COH process in the antagonist group patient (note: (1) *Indicates statistically significant difference (P < 0.05); (2) S: SOR group; C: control group; (3) n (SOR group) = 54, n (control group) = 54).

In the antagonist protocol group, the SOR group had a longer downregulation time and ovulation induction time than the control group, and the total amount of Gn used was more than that of the control group. The level of serum LH of the SOR group on day 2 of COH was 3.28 ± 2.22 IU/l, significantly lower than 4.79 ± 3.57 IU/l in the control group (P < 0.05). Conversely, the LH level on HCG day in the SOR group was 3.10 ± 2.50 IU/l, higher than 1.91 ± 1.16 IU/l in the control group (**Figure 5B**) (**Supplementary Table 3**). The level of LH/FSH ratio of the SOR group on day 2 of COH was 0.54 ± 0.30, also significantly lower than 0.87 ± 0.88 in the control group (P < 0.05) (**Supplementary Table 3**). The levels of serum E2 of the SOR group on COH days 6–8 and 9–11 were 430.19 ± 314.40 pmol/l and 1,170.88 ± 1,138.11 pmol/l, respectively, lower than 2,150.61 ± 2,790.17 pmol/l and 5,933.60 ± 4,309.70 pmol/l in the control group (**Figure 5A**). The level of serum P of the COH SOR group was 1.16 ± 0.47 nmol/l, lower than 1.88 ± 0.85 nmol/l in the control group on COH days 9–11 (P < 0.05) (**Figure 5C**). The PFI and P/E2 ratio in the SOR group on the HCG day were not statistically different (**Supplementary Table 3**). The minimum follicle number in the SOR group on days 6–8, 9–11, and 12–14 of COH was higher than that of the control group (12.28 ± 5.10 vs. 9.14 ± 5.80, 10.41 ± 5.68 vs. 5.81 ± 4.04, 9.50 ± 5.09 vs. 3.00 ± 1.00, respectively) (P < 0.05), and the number of large follicles in the SOR group on COH days 12–14 was 1.40 ± 0.70, significantly lower than 4.58 ± 2.28 in the control group (P < 0.05)(**Figure 6**). Other details are included in **Supplementary Table 3**.

##### 3.2.2.2 ROC Curve Analysis and Joint Diagnosis

In the univariate analysis, the data with statistical differences in the early and median follicular phases were analyzed using the ROC curve, according to the COH time sequence separately. The threshold of SOR occurrence and the corresponding sensitivity and specificity were calculated. The results are shown in **Table 3**. The level of LH and the LH/FSH ratio on COH day 2 were combined with BMI respectively for analysis, which resulted in a sensitivity of 77% and specificity of 72% (AUC, 0.758; 95% CI, 0.665–0.853) and sensitivity of 77% and specificity of 74% (AUC, 0.773; 95% CI, 0.681–0.865), respectively.

**Table 3:** Results of single-factor indexes during the COH process in the ultra-long/long group patients.

Time	Index	AUC	Threshold	Sensitivity	Specificity	95% confidence interval
BMI (kg/m^2^)		0.743	23.95	76%	68%	0.646–0.839
COH day 2	LH	0.685	2.47 IU/l	49%	85%	0.584–0.787
	LH/FSH ratio	0.675	0.57	64%	70%	0.573–0.777
COH days 9–11	Serum E2	0.912	3,372 pmol/l	100%	73%	0.822–1.000
	Serum P	0.773	1.62 nmol/l	88%	58%	0.633–0.913
	Number of minimum follicles	0.741	8.5	68%	81%	0.605–0.877
COH days 12–14	Number of minimum follicles	0.944	5	92%	100%	0.857–1.000
	Number of large follicles	0.929	2.5	90%	88%	0.844–1.000

### 3.3 Laboratory Outcome of patients in IVF

Among the three COH protocol groups, compared with the control group, the SOR group had no statistical difference (P > 0.05) in the number of oocytes obtained, fertilized oocytes, 2PN oocytes, cleavage, and high-quality embryos and the rate of 2PN and high-quality embryos (**Table 4**).

**Table 4:** Comparison of the laboratory outcomes of IVF patients.

Index	Ultra-long/long protocol			Antagonist protocol		
	SOR group (n = 71)	Control group (n = 71)	*P value*	SOR group (n = 54)	Control group (n = 54)	*P* value
Total number of oocytes obtained	13.46 ± 5.68	16.52 ± 8.95	0.157	13.91 ± 7.78	14.74 ± 9.96	0.595
Number of fertilized oocytes	12.63 ± 5.74	15.00 ± 8.69	0.261	12.24 ± 7.17	13.38 ± 9.34	0.437
Fertilization rate (%)	94.73 ± 14.20	91.36 ± 12.43	0.370	89.81 ± 16.92	92.20 ± 12.96	0.370
Number of 2PN oocytes	6.46 ± 3.53	6.96 ± 3.59	0.614	7.02 ± 5.54	8.50 ± 6.19	0.144
2PN rate (%)	51.94 ± 20.00	52.77 ± 26.39	0.901	56.76 ± 24.71	64.54 ± 22.16	0.348
Number of cleavage	8.21 ± 4.51	10.11 ± 6.72	0.247	9.08 ± 6.73	10.34 ± 7.04	0.285
Cleavage rate (%)	66.25 ± 24.83	67.91 ± 23.82	0.808	72.04 ± 29.29	78.42 ± 19.23	0.154
Number of high-quality embryos	4.83 ± 2.68	4.89 ± 3.08	0.946	4.98 ± 4.13	5.63 ± 4.23	0.362
High-quality embryo rate (%)	62.29 ± 23.88	51.30 ± 30.62	0.163	53.37 ± 22.53	57.63 ± 31.07	0.339

2PN rate = (number of 2PN oocytes/number of fertilized oocytes) *100%; Cleavage rate = (Number of cleavage/Number of fertilized oocytes) *100%; High-quality embryo rate = (Number of high-quality embryos/Number of cleavage) *100%

### 3.4 Clinical Pregnancy Outcome of patients in IVF

The cumulative live-birth rate of the SOR group (40.54%) in the antagonist protocol group was lower than that of the control group (79.36%); the difference was statistically significant (P < 0.05). Compared with the control group, the biochemical pregnancy rate, clinical pregnancy rate, live-birth rate, miscarriage rate, and full-term birth rate of the SOR group were not different from those of the control group (P > 0.05) (see **Supplementary Tables 4–7** for details).

## 4 Discussion

### 4.1 Signs of Patients with SOR

The results of this study showed that the occurrence of SOR has a correlation with patients’ higher BMI, which is consistent with previous studies (11). It is worth noting that the AMH of SOR patients is even higher than that of the control group in the ultra-long/long protocol group, indicating that AMH does not only stand for the ovarian reserve but could play roles in abnormal follicular formation (12, 13). Results also show that, in the antagonist group, the TSH level of SOR patients is lower than that of the control group, but both of them are within the normal range clinically, and thus will not affect following treatment.

Of note, our results showed that the serum E2 level during the COH process of the SOR group was lower than that of the control group. This could be caused by relatively insufficient E2 metabolism because of more adipose tissues in patients with obesity who had a larger body surface area, so that they need more Gn during the COH process to obtain mature follicles. Previous studies have shown (11) that patients who are overweight and obese have different degrees of metabolic disorders, such as insulin resistance and hyperleptinemia, which can affect oocyte maturation and embryonic development potential (14). Although some patients enrolled in this study were clinically overweight, we did not screen the level of metabolic indexes such as insulin, glucose tolerance, or blood lipid profiles for these patients because their previous assessments showed normal ovarian response. Therefore, we did not include the analysis about metabolic factors in this study.

### 4.2 Dynamics of Hormone Levels During the COH Process

#### 4.2.1 Analysis of the LH/FSH Ratio

Our study showed a lower basic LH/FSH ratio at the beginning of the COH process in SOR patients compared with the control group (basic LH/FSH ratio in ultra-long/long protocol, LH/FSH ratio on COH day 2 in antagonist protocol, respectively). A small section of the population could be more sensitive to downregulation, exhibiting excessive pituitary suppression and insufficient LH levels (4, 15). Usually, GnRH agonists inhibit 90% of the LH level but only inhibit about 40%–50% of the FSH level. The residual LH in the serum of most patients after downregulation is sufficient to support the development of multiple follicles in most cases; however, differences may exist in the sensitivity of the adenohypophysis to GnRH-a among different patients, for whom the relative or absolute lack of LH after pituitary downregulation could be the main reason for SOR when the long protocol is used (16).

#### 4.2.2 Analysis of E2 and P

Our results indicated that during the process of COH, the levels of E2 and P in each protocol group of the SOR group were lower than those of the control group. The relatively lower E2 is mainly manifested in the early follicular phase, whereas the lower serum P is mainly manifested in the late follicular phase. This reason of lower E2 lies in that, in the early follicular phase, LH can stimulate the theca cells (TCs) to synthesize androgens, which are substrates for estrogen synthesis, and estrogen plays an important role in follicular growth and development. When the downregulation is too deep and the level of serum LH is too low, androgen synthesis will be insufficient, leading to a poor increase in serum E2 level and follicular development stagnation.

With regard to lower serum P, of note, it mainly happened at the late follicular phase in our study. Progesterone in the early follicular phase is mainly secreted by the adrenal glands, whereas progesterone in the late follicular phase is mainly derived from the ovaries, and this steroidal conversion by the adrenal gland is regulated by the ovaries (17). GCs express small amounts of LHR on the cell surface and respond to LH in the follicular stage; when multiple follicles mature, the number of luteinized GCs increases, which accordingly causes synthetic progesterone to gradually increase within 12–24 h before the appearance of the LH peak (18). With relatively insufficiency of LH, GCs synthesize less progesterone, as manifested in our study.

#### 4.2.3 Analysis of the P/E2 Ratio and PFI

Previous studies have shown that a high PFI (18, 19) and a higher P/E2 ratio (20) each on the HCG day are independent risk factors for the reduction of live-birth rate in long protocol: for individuals with normal ovarian responses and high ovarian response, P/E2 ≥0.48 (unit of P, ng/ml) and ≥0.42, respectively, the live-birth rate is significantly reduced. In our study, the P/E2 ratio on the HCG day in each protocol group showed a higher trend in the SOR group than in the control group, but without significant statistical difference, which may be related to our limited sample size, and SOR has been recognized and the poor follicular development has been rescued to some extent by experienced clinicians. Further prospective study with a larger sample size is needed to verify the efficiency of P/E2 ratio on HCG day to predict the clinical outcome for SOR patients.

### 4.3 Follicle Growth During the COH Process

In the ultra-long/long protocol group, the minimum follicle number in the SOR group on COH days 6–8 was more than that in the control group, and this difference persisted until COH days 12–14, whereas the number of relatively larger follicles was lower than that in the control group, which is shown by the fact that in the cross section of each monitoring time, the retardation of follicular development persists and gradually accumulates.

Notably, the number of large follicles in the SOR group in the late follicular phase was lower than that in the control group in both protocol groups, but statistical difference only showed in the ultra-long/long protocol group. It might be caused by our limitation to a single center and small sample size but more likely due to the pituitary downregulation treatment in the ultra-long/long protocol, because correction of the excessively deep pituitary inhibition state through the adjustment of Gn in the later stage is more difficult in this group, resulting in the difficulty for the final number of mature follicles to reach the normal level, whereas for patients in the antagonist protocol group, Gn is activated without pre-pituitary downregulation.

### 4.4 Laboratory Outcome and Clinical Outcome

There is no significant difference in laboratory outcomes between SOR patients and control patients in both protocol groups, possibly due to the prolonged use of Gn because clinicians in our reproductive center identified SOR in the early stage of the COH process and added r-LH/HMG or prolonged the time frame of treatment to revert SOR to a normal response. Early identification and remedial measures are essential to transforming SOR to a normal response to obtain good clinical outcomes.

Lower fresh-cycle live-birth rates of the SOR group were recorded in the ultra-long/long protocol group (P <0.05), and lower cumulative-cycle live-birth rates of the SOR group were recorded in the antagonist protocol group (P <0.05), indicating that the occurrence of SOR has an adverse effect on pregnancy outcome, although no significant difference of laboratory outcomes between SOR patients and control group. Because of technical limitation, we can only asses the outcome of COH referring to the number of oocytes retrieved and the morphology of oocytes and embryos, but not the quality of the oocytes in in essence (8). On the other hand, Gn dosage and Gn days for SOR patients are significantly higher than in the control group (**Supplementary Tables 2, 3**); possibilities remain that prolonged Gn exposure may detrimentally affect endometrial receptivity (21, 22).

### 4.5 Clinical Features in the Early Follicular Phase

Recognizing that SOR in time is important clinically, in most cases sensitivity has greater clinical significance than specificity; clinicians should at least detect patients at higher risk first and distinguish real SOR patients. A comprehensive evaluation of these indicators can improve the detection sensitivity of SOR.

Based on our results, for patients who adopt the ultra-long/long protocol, when the basic LH/FSH ratio <0.61 and the BMI >21.35 kg/m^2^, the patient is more likely to have SOR, and clinicians need to have a high index of suspicion to deal with such cases in time. In the follow-up period of follicular monitoring, when the minimum follicle number on COH days 9–11 >10.5, number of small follicles on COH days 9–11 <3.5, minimum follicle number on COH days 12–14 >5.5, or number of medium follicles <2.5, and serum P <1.52 nmol/l, the patient may have already experienced SOR at this time, and the clinician should be able to manage. COH days 12–14 belong to the middle follicular phase; therefore, if not treated in time, SOR may not be rescued.

For patients undergoing the antagonist protocol, when LH on COH day 2 <2.47 IU/l and BMI >21.35 kg/m^2^, or the LH/FSH ratio on COH day 2 <0.57 and BMI >21.35 kg/m^2^, SOR may possibly occur, and clinicians should have a high index of suspicion to detect and manage the patient early. In the follow-up follicle monitoring period, COH days 9–11, serum E2 <3,372.0 pmol/l, serum P < 1.62 nmol/l, minimum follicle number >8.5, minimum follicle number on COH days 12–14 >5, or number of large follicles <2.5 indicates that the patient has already experienced SOR at this time and needs to be treated in time; otherwise, SOR may not be rescued.

Due to the limitation of sample size, this study did not separately calculate the indicators of the ultra-long protocol and long protocol. Therefore, the results are limited in clinical application, but they serve as reference and foundation for future studies with larger sample sizes.

### 4.6 Treatment of patients with SOR

#### 4.6.1 Early Recognition of SOR

Based on our study, some points should be emphasized. In treating patients with SOR, first, an experienced doctor should comprehensively and accurately assess the patient’s general condition to the greatest extent. Previous history of SOR or even a poor COH outcome and all possible related risk factors of SOR should be brought to the forefront, including general condition such as BMI, previous IVF records, ovarian reserve, uterine condition, and basal serum hormone condition, to assess the function of the hypothalamic–pituitary–ovarian (HPO) axis. Patients with obesity should be recommended to lose weight (23).

Next, during the COH process, clinicians should carefully observe the patient’s follicular growth, serum E2 level, and COH time to identify abnormal growth conditions. Here based on our analysis, some threshold values of some clinical indexes including BMI and levels of serum sex hormones are provided to be taken as reference for the early recognition of SOR.

#### 4.6.2 Supplement of Gn

In our reproductive center, Gn dosage was calculated according to the estimated ovarian function based on clinical routines and adjusted during the COH process according to the experience of clinicians. In most cases, the application of FSH should be sufficient, and even if insufficient, the monitoring doctors make adjustments to supplement the FSH dosage when patients first return to the hospital on days 4–6 following FSH severing; therefore, the majority of patients have good outcomes. However, SOR still occurs, indicating the complexity of SOR. We could preliminarily divide the possible causes of SOR into the following categories: 1) too deep pituitary function suppression by GnRH-a; 2) different sensitivity to Gn in the patient population, some of which might be explained by FSH receptor and/or LH receptor gene polymorphism (24) (20); 3) clinicians are unaware of the occurrence of SOR and do not supplement enough Gn in time. Of note, the GnRH antagonist protocol does not require downregulation pretreatment. Previous studies indicated that the incidence of SOR on the antagonist protocol was 4.8%, which was lower than that of the long protocol (15). However, it was a single-center study and the sample size of the SOR group in this study was limited (<100). The mechanism of occurrence of SOR in the antagonist protocol is more likely to be related with LH receptor mutation or an insensitive LH receptor (25).

Currently, receptor polymorphism inspection is not able to be widely used in clinic because it is time-consuming and money-consuming for most patients; of course, for patients with unexplained refractory SOR, FSH/LH receptor gene polymorphism inspection should be suggested to patients. Half-dose long-acting injection of GnRH-a or even 1/4~1/3 dosage can be considered to prevent excessive suppression of the pituitary gland for some patients with a history of SOR (1, 4). When serum LH <1.0 IU/l, the startup time of Gn can be considered for postponement or that LH can be used at the startup time.

In most cases, supplement of Gn is still the main method. Simply increasing FSH cannot improve the lack of endogenous LH (26); either LH or HMG is more efficient. Recombinant human luteinizing hormone (r-hLH) can highly mimic the biological function of LH *in vivo* (7, 27, 28); LH in the early follicular phase can act on TCs, increasing the production of E2 from GCs by promoting androgen synthesis and enhancing the sensitivity of GCs to FSH to improve ovarian responsiveness (29, 30). Moreover, the number of oocytes retrieved and the mature oocyte percentage obtained by adding 150 LH are higher than those added to 75 U (26); Jing et al. (31) revealed that when HMG (containing both FSH and LH) at 75 U/day was added during the early follicular phase (COH days 4–8), the number of oocytes obtained, number of available embryos, implantation rate, and clinical pregnancy rate were apparently higher than when it was added during the late follicular phase. Some studies (24) also show that in patients with SOR on the long protocol, adding HMG with a growth hormone (GH) at 4.5 IU/day until the HCG day is more effective than adding HMG only.

#### 4.6.3 Monitor Development of Follicles

Patients with suspected SOR should be evaluated for FSH dose sufficiency, follicular growth rate, and E2 rate of increase, to assess whether LH is sufficient. Since LH is secreted in pulses, the detected serum LH level during the COH process does not completely represent the actual LH level *in vivo*; relying solely on the serum LH level to assess the occurrence or prediction of SOR may not be beneficial. Serum E2 and follicular growth are still the main focus during the COH monitoring process; if follicular growth and E2 rise show an upward trend, the time of Gn use can be continued to ensure that the oocytes fully mature. Otherwise, LH or HMG should be added in time.

Our study indicates that SOR has adverse effects on clinical outcome. Early recognition of SOR can be of great importance for rescuing it. Specific treatment protocol for SOR needs to be clarified by more detailed prospective controlled studies.

## Data Availability Statement

The original contributions presented in the study are included in the article/**Supplementary Material**. Further inquiries can be directed to the corresponding author.

## Ethics Statement

The studies involving human participants were reviewed and approved by the Ethics Committee of Peking University Third Hospital (No. IRB00006761-M2020004). Informed consent was waived because this was a data analysis with no personally identified information. Written informed consent for participation was not required for this study in accordance with the national legislation and the institutional requirements.

## Author Contributions

YY performed the literature search, collected and analyzed the data, and wrote the manuscript; RQ, XM, and SQ helped screening the medical records and collecting the data; XN and LC helped analyzing the data; RY, YW, RL, and JQ critically reviewed and revised the manuscript. All authors read and approved the final submission.

## Funding

This work was supported by National Natural Science Foundation of China [81873833], National Natural Science Foundation of China [81550022], and Key Clinical Research Project of Peking University Third Hospital (BYSY2018016).

## Conflict of Interest

The authors declare that the research was conducted in the absence of any commercial or financial relationships that could be construed as a potential conflict of interest.

## Publisher’s Note

All claims expressed in this article are solely those of the authors and do not necessarily represent those of their affiliated organizations, or those of the publisher, the editors and the reviewers. Any product that may be evaluated in this article, or claim that may be made by its manufacturer, is not guaranteed or endorsed by the publisher.
