# Supplementary material for: Clinical Features and Management of Suboptimal Ovarian Response During in vitro Fertilization and Embryo Transfer: Analysis Based on a Retrospective Cohort Study
Source: Front Endocrinol (Lausanne). 2022 Jul 22;13:938926. doi: 10.3389/fendo.2022.938926 (PMC10204704; doi:10.3389/fendo.2022.938926)
Supplement: Supplementary file 1 [file DataSheet_1.docx]

Supplementary Material

# Supplementary Data

**Appendix E1. Treating process of each protocol**

## Long protocol:

GnRH-a (Diphereline or Enantone 1.8mg) was started from the previous midluteal stage after pregnancy was excluded. After 14-21 days, the pituitary gland reached downregulation state(Standards: LH<5 IU/L, E2<200pmol/L , Endometrium thickness<5mm), and then exogenous gonadotropins(Gn) 150-450IU/d were used, including recombinant FSH (Gonal-f; Merck Serono, Geneva, Switzerland and Purigon, Organon, P.O. Box 20 OssNL5340BH, Netherlands) or Urofollitropin(Urofollitropin for Injection, (Lishenbao), 75IU, Livzon Pharmaceutical Group, Zhuhai, China),urine-derived HMG (injection gonadotropin, 75U, Livzon Pharmaceutical Group, Zhuhai, China)/high-purity urine-derived HMG (Menopur, 75IU, Ferring GmbH), recombinant LH (Luveris, 75IU, Merck Serono S.A), etc. to promote ovulation. Amount of Gn was be adjusted during COH process according to the follicular growth situation and level of serum E2. When the diameter of three dominant follicles reached or exceeded 18 mm, 250 ug of recombinant hCG (Merck Serono SA Aubonne Branch) was injected, and the oocytes were retrieved 36 hours later.

## Ultra-long protocol:

GnRH-a 3.75mg was injected once at the early stage of follicular phase after pregnancy was excluded. 28-35 days later, serum FSH, LH, E2, P levels were tested, and ultrasound was performed to monitor the follicle development. After achieving sufficient pituitary down-regulation, Gn 150-450 IU/d was be used, the types of Gn drugs were the same as above. Amount of Gn would be adjusted during COH process according to the follicular growth situation and level of serum E2. When the 3 dominant follicles reached or exceeded 18mm, 250ug of recombinant hCG (same as above) was injected, and oocytes were retrieved 36 hours later.

## Antagonist protocol:

On the second day of menstruation when no large follicles in the ovaries confirmed by ultrasound and the serum hormones levels were in the basic state, Gn was started to be used to induce ovulation, drug types were the same as before. Amount of Gn would be adjusted during COH process according to the follicular growth situation and level of serum E2. There were 2 options for the timing of Gn administration: 1)Fixed administration program: antagonist was added on the 5th to 7th days after Gn administration; 2)Flexible administration protocol: antagonist(Cetrorelix acetate, Pierre Fabre Pharmaceuticals or Ganirek, 250ug, N.V.Organon) was added according to the size of follicles and serum LH level, generally when the diameters of dominant follicle reach 14mm or LH≥10IU/L. When the diameters of three dominant follicles reached or exceeded 18mm, 250ug of recombinant hCG(same as above) was injected, and the oocytes were retrieved 36 hours later.

## Appendix E2. Standards for pituitary regulation

## 1) Endometrial thickness ≤5mm;

2) No large follicles or cysts in bilateral ovaries confirmed by ultrasound: follicular diameter<9mm) ;

3)serum LH<5IU/L;

4) serum E2<200pmol/l.

# Supplementary Figures and Tables

## Supplementary Tables

Supplementary Table 1. General information of Ultra-long and long group

| Index | SOR group | | | Control group | | |
| --- | --- | --- | --- | --- | --- | --- |
|  | Ultra-long protocol（n=24） | Long protocol（n=47） | P value | Ultra-long protocol（n=24） | Long protocol（n=47） | P value |
| age | 29.83±2.70 | 29.60±3.08 | 0.750 | 30.93±3.10 | 29.93±3.27 | 0.191 |
| Spouse age | 31.33±2.93 | 30.30±2.83 | 0.154 | 31.11±2.87 | 30.76±3.06 | 0.624 |
| Infertility year | 3.00±2.08 | 2.85±2.31 | 0.792 | 3.48±2.19 | 2.85±1.88 | 0.183 |
| BMI  (kg/m2) | 24.66±3.33 | 24.79±3.58 | 0.884 | 21.68±2.68 | 21.62±3.09 | 0.933 |
| Basal FSH(IU/L) | 6.43±1.57 | 6.61±2.76 | 0.806 | 5.52±1.76 | 6.35±2.07 | 0.107 |
| Basal LH(IU/L) | 4.07±2.63 | 3.87±2.26 | 0.768 | 4.17±2.58 | 4.74±2.73 | 0.412 |
| Basal E2(pmol/L) | 152.14±76.53 | 176.36±119.59 | 0.453 | 188.15±71.28 | 158.48±58.17 | 0.067 |
| Basal PRL(ng/ml) | 29.23±62.42 | 22.37±38.18 | 0.607 | 50.25±135.67 | 49.63±50.06 | 0.271 |
| Basal T(nmol/L) | 4.99±15.86 | 2.14±5.68 | 0.521 | 2.04±5.79 | 1.27±3.56 | 0.494 |
| Basal AND  (nmol/L) | 6.08±2.27 | 7.53±3.79 | 0.199 | 5.80±2.91 | 6.16±2.80 | 0.641 |
| AMH  (ng/ml) | 4.54±2.67 | 4.89±3.50 | 0.732 | 3.27±2.15 | 3.58±2.62 | 0.606 |
| TSH  (uIU/ml) | 2.80±1.58 | 2.44±1.80 | 0.468 | 2.65±1.64 | 2.23±1.29 | 0.205 |
| AFC  (bilateral) | 12.22±4.42 | 13.96±5.02 | 0.162 | 12.26±4.97 | 11.74±5.83 | 0.710 |
| Gn days | 17.13±2.59 | 17.72±2.59 | 0.361 | 12.11±1.31 | 11.89±2.01 | 0.607 |
| Gn dosage（IU） | 4439.58±1139.81 | 4145.48±1026.76 | 0.470 | 2637.50±1006.81 | 2676.71±795.49 | 0.849 |

Supplementary Table 2. Comparison of indexes during COH process in ultra-long/long group

| Index | SOR group（n=71） | | | control group（n=71） | *P* value | |
| --- | --- | --- | --- | --- | --- | --- |
| Gn days | 17.52±2.59 | | | 12.15±1.14 | <0.05 | |
| Gn dosage（IU） | 4211.10±1062.17 | | | 2685.81±834.50 | <0.05 | |
| endometrial thickness（mm） | 11.44±2.05 | | | 10.74±1.84 | 0.103 | |
| Basal LH/FSH ratio | 0.63±0.37 | | | 0.84±0.65 | <0.05 | |
| COH2nd day FSH（IU/L） | 2.38±1.61 | | | 2.16±1.10 | 0.362 | |
| COH2nd LH（IU/L） | 1.34±1.16 | | | 1.18±0.78 | 0.330 | |
| COH2nd E2（pmol/L） | 160.57±291.02 | | | 121.57±44.37 | 0.270 | |
| COH2nd P（nmol/L） | 1.05±0.45 | | | 1.07±0.56 | 0.785 | |
| COH2nd LH/FSH ratio | 0.76±1.13 | | | 0.69±0.54 | 0.628 | |
| COH6-8th day E2（pmol/L） | 231.85±108.58 | | | 1007.58±1002.80 | <0.05 | |
| COH12-14th day E2（pmol/L） | 1342.95±1030.48 | | | 6839.12±4183.28 | 0.141 | |
| HCG day E2（pmol/L） | 9177.45±5476.03 | | | 7832.64±6031.53 | 0.293 | |
| COH6-8th day LH（IU/L） | 0.71±0.47 | | | 0.74±0.46 | 0.712 | |
| COH12-14th day LH（IU/L） | 0.65±0.43 | | | 0.79±0.43 | 0.272 | |
| HCG day LH（IU/L） | 0.75±0.90 | | | 0.52±0.47 | 0.298 | |
| COH6-8thday P（nmol/L） | | 1.00±0.52 | 1.12±0.57 | | 0.453 |  |
| COH12-14th day P（nmol/L） | | 1.09±0.55 | 2.02±1.11 | | <0.05 |  |
| HCG day P（nmol/L） | | 2.18±1.01 | 2.58±1.26 | | 0.154 |  |
| COH6-8thday number of minimum follicles* | | 11.78±4.29 | 9.86±4.63 | | <0.05 |  |
| COH6-8thday number of small follicles | | 3.25±2.06 | 4.17±4.38 | | 0.684 |  |
| COH9-11thday number of minimum follicles | | 12.42±5.15 | 6.24±3.02 | | <0.05 |  |
| COH9-11thday number of small follicles* | | 3.75±3.26 | 8.42±3.85 | | <0.05 |  |
| COH12-14^th^ day number of minimum follicles | | 10.49±4.91 | 3.00±1.41 | | <0.05 |  |
| COH12-14^th^ day number of small follicles | | 6.12±4.48 | 6.18±3.05 | | 0.930 |  |
| COH12-14^th^ day number of medium follicles* | | 2.89±1.90 | 5.60±3.53 | | <0.05 |  |
| COH15-17^th^ day number of small follicles | | 7.03±4.21 | 4.75±1.73 | | <0.05 |  |
| COH15-17^th^ day number of medium follicles | | 5.31±2.91 | 4.64±2.46 | | 0.347 |  |
| COH15-17^th^ day number of large follicles* | | 3.24±2.09 | 5.27±1.49 | | <0.05 |  |
| HCG day PFI | | 0.59±0.25 | 0.56±0.41 | | 0.650 |  |
| HCG day P/E2（×10^-4^） | | 0.33±0.24 | 0.12±0.68 | | 0.279 |  |

Supplementary Table 3. Comparison of indexes during COH process in antagonist group

| Index | | SOR group  （n=54） | | control group  （n=54） | | *P* value | |
| --- | --- | --- | --- | --- | --- | --- | --- |
| Gn days | | 15.39±1.84 | | 10.30±1.38 | | <0.05 | |
| Gn dosage(IU) | | 3656.48±1073.83 | | 1871.76±661.40 | | <0.05 | |
| endometrial thickness（mm） | | 5.57±1.29 | | 6.05±1.72 | | 0.112 | |
| Basal LH/FSH ratio | | 0.91±0.87 | | 1.00±0.59 | | 0.574 | |
| COH2nd day FSH（IU/L） | | 6.04±1.93 | | 6.22±2.19 | | 0.653 | |
| COH2nd day LH（IU/L） | | 3.28±2.22 | | 4.79±3.57 | | <0.05 | |
| COH2nd day E2（pmol/L） | | 168.72±57.19 | | 176.79±61.06 | | 0.482 | |
| COH2nd day P（nmol/L） | | 1.20±0.67 | | 1.48±1.04 | | 0.116 | |
| COH2nd day LH/FSH ratio | | 0.54±0.30 | | 0.87±0.88 | | <0.05 | |
| COH6-8th day  serum E2（pmol/L） | | 430.19±314.40 | | 2150.61±2790.17 | | <0.05 | |
| COH9-11th day  serum E2（pmol/L） | | 1170.88±1138.11 | | 5933.60±4309.70 | | <0.05 | |
| COH12-14th day  serum E2（pmol/L） | | 3365.92±2673.04 | | 8480.57±7100.94 | | 0.107 | |
| HCG day  serum E2（pmol/L） | | 9380.13±6651.09 | | 9484.98±6192.02 | | 0.666 | |
| COH6-8th day  serum LH（IU/L） | | 2.45±1.92 | | 2.51±2.35 | | 0.885 | |
| COH9-11th day  serum LH（IU/L） | | 2.49±2.29 | | 2.25±1.44 | | 0.678 | |
| COH12-14th day  serum LH（IU/L） | | 2.08±1.00 | | 1.86±1.07 | | 0.609 | |
| HCG day  serum LH（IU/L） | | 3.10±2.50 | | 1.91±1.16 | | <0.05 | |
| COH6-8th day  serum P（nmol/L） | | 0.99±0.42 | | 1.10±0.61 | | 0.065 | |
| COH9-11th day  serum P（nmol/L） | | 1.16±0.47 | | 1.88±0.85 | | <0.05 | |
| COH12-14th day  serum P（nmol/L） | | 1.37±0.66 | | 2.38±1.32 | | 0.095 | |
| HCG day  serum P（nmol/L） | | 2.59±1.33 | | 2.39±0.99 | | 0.374 | |
| COH6-8th day number of minimum follicles | | 12.28±5.10 | | 9.14±5.80 | | <0.05 | |
| COH6-8th day number of small follicles | | 4.13±2.87 | | 6.30±4.95 | | 0.105 | |
| COH9-11th day number of minimum follicles | | 10.41±5.68 | | 5.81±4.04 | | <0.05 | |
| COH9-11th number of small follicles | | 5.28±4.41 | | 6.13±3.80 | | 0.318 | |
| COH9-11th day number of medium follicles | | 2.25±0.96 | | 4.83±3.12 | | 0.109 | |
| COH12-14th day number of minimum follicles | | 9.50±5.09 | | 3.00±1.00 | | <0.05 | |
| COH12-14th day number of small follicles | | 7.27±4.32 | | 6.50±4.18 | | 0.473 | |
| COH12-14th day number of medium follicles | | 3.24±2.29 | | 4.08±2.40 | | 0.195 | |
| COH12-14th day number of large follicles | | 1.40±0.70 | | 4.58±2.28 | | <0.05 | |
| COH15-17th day number of small follicles | | 6.06±3.65 | | 11.00±8.18 | | 0.406 | |
| COH15-17th day number of medium follicles | | 5.04±3.02 | | 6.00±1.41 | | 0.661 | |
| COH15-17th day number of large follicles | | 3.81±1.86 | | 4.00±2.00 | | 0.868 | |
| HCG day P/number of large follicles（PFI） | | 0.71±0.39 | | 0.65±0.66 | | 0.564 | |
| HCG day P/E2（×10-4） | | 3.60±2.14 | | 3.06±1.68 | | 0.153 | |

Supplementary Table 4. Comparison of clinical outcomes of fresh cycle in COH of ultra-long/long protocol

| Index | SOR group  （n=71） | Control group  (n=71) | X2 value | *P* value |
| --- | --- | --- | --- | --- |
| Biochemical pregnancy rate(%) | 43.75 | 56.25 | 0.500 | 0.480 |
| Clinical pregnancy rate(%) | 37.50 | 50.00 | 0.508 | 0.467 |
| Live-birth rate(%) | 33.33 | 87.50 | 4.381 | <0.05 |
| Miscarriage rate (%) | 50.00 | 12.50 | 2.363 | 0.124 |
| Full-term birth rate(%) | 33.33 | 75.00 | 2.431 | 0.119 |

Supplementary Table 5. Comparison of clinical outcomes of cumulative cycle in COH of ultra-long/long protocol

| Index | SOR  group  （n=71） | control group  (n=71) | X2 value | *P* value |
| --- | --- | --- | --- | --- |
| Cumulative biochemical pregnancy rate(%) | 58.33 | 46.67 | 0.894 | 0.344 |
| Cumulative clinical pregnancy rate(%) | 50.00 | 40.00 | 0.660 | 0.417 |
| Cumulative live-birth rate(%) | 55.56 | 83.33 | 2.50 | 0.114 |
| Cumulative miscarriage rate (%) | 33.33 | 8.33 | 2.52 | 0.113 |
| Cumulative full-term birth rate(%) | 55.56 | 75.00 | 1.17 | 0.279 |

Note:

Cumulative biochemical pregnancy rate: In one same oocytes retrieval cycle, the total number of biochemical pregnancies(both fresh embryos and frozen embryos transplanted)/the number of all transplant cycles

Cumulative clinical pregnancy rate: In one same oocytes retrieval cycle, the total number of clinical pregnancies(both fresh embryos and frozen embryos transplanted)/the number of all transplant cycles

Cumulative live-birth rate: In one same oocytes retrieval cycle, the total number of live-birth(both fresh embryos and frozen embryos transplanted)/the number of all clinical pregnancies

Cumulative miscarriage rate: In one same oocytes retrieval cycle, the total number of miscarriage(both fresh embryos and frozen embryos transplanted)/the number of all clinical pregnancies

Cumulative full-term birth rate: In one same oocytes retrieval cycle, the total number of full-term birth(both fresh embryos and frozen embryos transplanted)/the number of all clinical pregnancies

Supplementary Table 6. Comparison of clinical outcomes of fresh cycle in COH of antagonist protocol

| Index | SOR group  （n=54） | control group  (n=54) | X2 value | *P* value |
| --- | --- | --- | --- | --- |
| Biochemical pregnancy rate(%) | 51.35 | 41.79 | 0.880 | 0.348 |
| Clinical pregnancy rate(%) | 51.35 | 34.33 | 2.869 | 0.090 |
| Live-birth rate(%) | 78.95 | 73.91 | 0.145 | 0.703 |
| Miscarriage rate (%) | 10.53 | 4.35 | 0.599 | 0.439 |
| Full-term birth rate(%) | 63.16 | 65.22 | 0.019 | 0.890 |

Supplementary Table 7. Comparison of clinical outcomes of cumulative cycle in COH of antagonist protocol

| Index | SOR  group  （n=54） | control group  (n=54) | X2 value | *P* value |
| --- | --- | --- | --- | --- |
| Cumulative biochemical pregnancy rate(%) | 55.41 | 48.67 | 0.90 | 0.343 |
| Cumulative clinical pregnancy rate(%) | 50 | 42 | 1.28 | 0.257 |
| Cumulative live-birth rate(%) | 40.54 | 79.36 | 15.44 | <0.05 |
| Cumulative miscarriage rate (%) | 18.92 | 7.94 | 2.66 | 0.103 |
| Cumulative full-term birth rate(%) | 62.16 | 71.43 | 0.92 | 0.338 |
